# NXP032 ameliorates cognitive impairment by alleviating the neurovascular aging process in aged mouse brain

**DOI:** 10.1038/s41598-023-35833-x

**Published:** 2023-05-26

**Authors:** Jae-Min Lee, Joo Hee Lee, So Hee Kim, Tae Hyeok Sim, Youn-Jung Kim

**Affiliations:** 1 College of Nursing Science, Kyung Hee University, Seoul, 02447 Republic of Korea; 2 Korea Armed Forces Nursing Academy, Daejeon, 34059 Republic of Korea; 3 Department of Nursing, Graduate School, Kyung Hee University, Seoul, 02447 Republic of Korea

**Keywords:** Cognitive ageing, Cognitive neuroscience

## Abstract

Vascular aging is well known to be associated with the breakdown of the neurovascular unit (NVU), which is essential for maintaining brain homeostasis and linked to higher cognitive dysfunction. Oxidative stress is believed to be a significant cause of the vascular aging process. Vitamin C is easily oxidized under physiological conditions, so it loses its potent antioxidant activity. We developed a DNA aptamer that enhances the function of vitamin C. NXP032 is the binding form of the aptamer and vitamin C. In this study, we investigated the effect of NXP032 on neurovascular stabilization through the changes of PECAM-1, PDGFR-β, ZO-1, laminin, and glial cells involved in maintaining the integrity of the blood–brain barrier (BBB) in aged mice. NXP032 was orally administered daily for 8 weeks. Compared to young mice and NXP032-treated mice, 20-month-old mice displayed cognitive impairments in Y-maze and passive avoidance tests. NXP032 treatment contributed to reducing the BBB damage by attenuating the fragmentation of microvessels and reducing PDGFR-β, ZO-1, and laminin expression, thereby mitigating astrocytes and microglia activation during normal aging. Based on the results, we suggest that NXP032 reduces vascular aging and may be a novel intervention for aging-induced cognitive impairment.

## Introduction

Aging is a natural and complicated process characterized by a progressive decline in physiological integrity, leading to impaired function and increased risk of disease. Oxidative stress is one of the significant contributors to the aging process. When there is an imbalance between the production of reactive oxygen species (ROS) and the body's antioxidant defense system, it can lead to oxidative damage to the cells of the neurovascular unit (NVU) and neuroinflammation^1^. This can result in decreased blood flow to the brain, which can contribute to cognitive decline and neurodegenerative diseases such as Alzheimer's disease, Parkinson’s disease, stroke, and vascular dementia^2^.

The NVU consists of endothelial cells, pericytes, astrocytes, microglia, neuron, and the basal lamina, which work together to maintain the integrity of the blood–brain barrier (BBB) and regulate cerebral blood flow. The BBB helps to maintain the homeostasis of the brain environment by restricting the passage of harmful substances and pathogens into the brain^3^. The breakdown of functional components of the BBB can occur with the normal aging process, even without cognitive decline and neurological disorders^4^. The BBB is composed of specialized endothelial cells tightly connected to each other by tight junction proteins. Age-related changes in the tight junction proteins can lead to increased BBB permeability and can increase the infiltration of immune cells and inflammatory mediators into the brain, leading to chronic neuroinflammation^5^. The levels of tight junction protein zonula occludens-1 (ZO-1) are dynamic and respond to physiological and pathological conditions, including hypoxia, oxidative stress, and inflammation, which are features of normal aging and could be driving BBB leakage^6–8^. Decreased pericyte coverage and function can also increase neuroinflammation^9^. In normal aging, BBB disruption is exacerbated when exposed to sequential insults such as neuroinflammation. BBB disruption may be an early event in the aging process, which can lead to neuroinflammation^10^. Chronic neuroinflammation, in turn, can exacerbate BBB dysfunction^11^. Both BBB dysfunction and neuroinflammation are thought to contribute to age-related cognitive decline and the development of age-related neurological disorders^12^.

Overall, maintaining BBB integrity is critical for healthy aging, as it helps to protect the brain from the damaging effects of inflammation and oxidative stress. By reducing oxidative stress and protecting the BBB, it may be possible to promote healthy aging and reduce the risk of age-related neurological disorders. Antioxidants can contribute to being protective against aging and age-related diseases by reducing oxidative stress. Ascorbic acid (vitamin C) is a potent antioxidant in biological systems that can combat oxidative stress. However, ascorbic acid loses its potency easily through oxidation. We have developed Aptamin® C320, a DNA aptamer that specifically binds to vitamin C and inhibits its oxidation. Aptamers are single-stranded DNA-based oligonucleotides, and Aptamin® C320 inhibits the oxidation of vitamin C and preserves its antioxidant activity in the body^13^. NXP032, a complex of vitamin C and Aptamin® C320, effectively removes ROS and increases antioxidant enzyme activity. It maintains a stable antioxidant effect by inhibiting oxidative stress induced by the activation of the antioxidant response element (ARE) pathway in aged mice^14^.

The aim of this study was to assess the potential neuroprotective effects of NXP032, a vitamin C/DNA aptamer complex, on age-related cognitive impairment. Specifically, the study aims to investigate whether NXP032 can mitigate the negative impact of aging on the brain by reducing the BBB damage and neuroinflammation in aged mice.

## Results

### NXP032 improves memory impairment in aged mice

**Figure 1:**
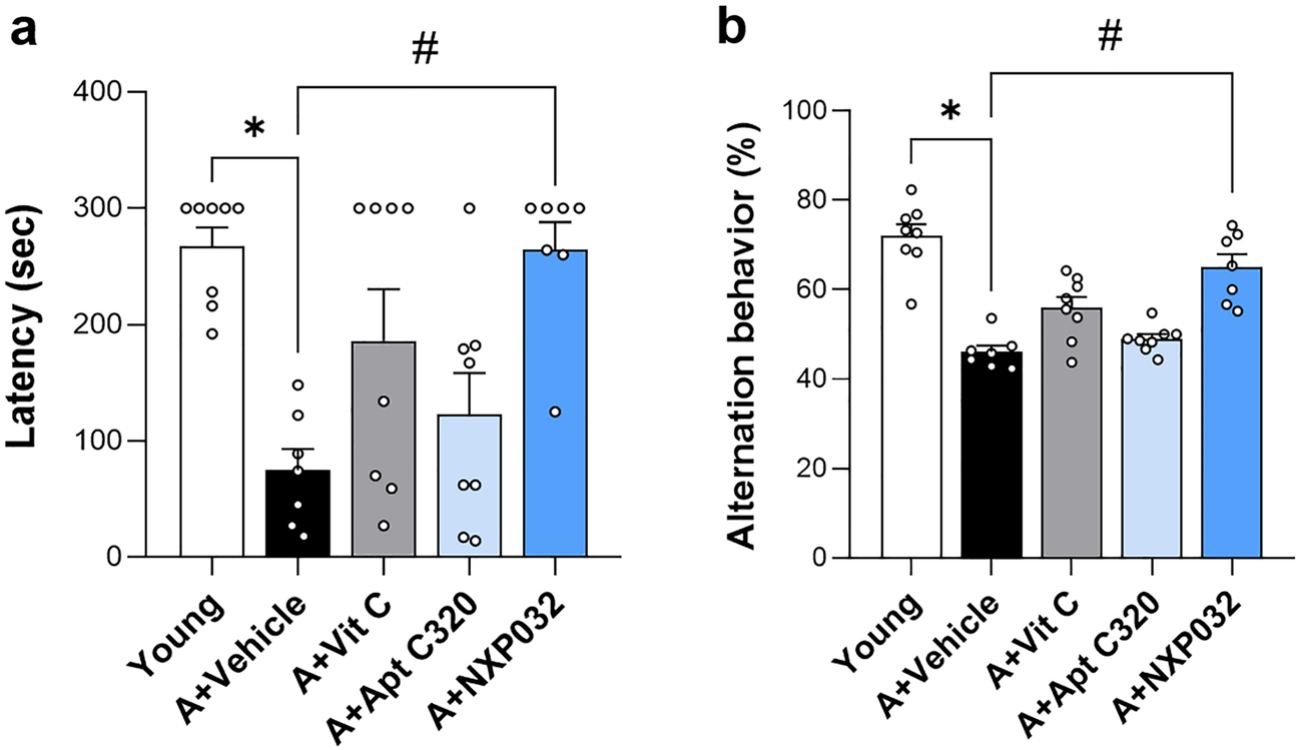
NXP032 improves cognitive function in aged mice. (**a**) In the passive avoidance test, NXP032 treatment in aged mice increased latency times compared to vehicle treatment in aged mice. (**b**) In the Y-maze test, NXP032 treatment significantly increased spontaneous alternation behavior compared to vehicle treatment. Data represent the mean ± standard error of the mean. *p < 0.05, compared to the young group. ^#^*p* < 0.05, compared to the A + Vehicle group.

We performed the passive avoidance and Y-maze tests to confirm the effects of NXP032 administration on cognitive function (Fig. 1). The passive avoidance test was used to assess learning and memory based on negative reinforcement^15^. A one-way ANOVA revealed a significant difference in the passive avoidance and Y-maze tests among the five groups (F = 4.748,* p* < 0.01; F = 23.223, *p* < 0.001). The 20-month-old mice exhibited memory impairment compared to the 4-month-old mice, as evidenced by significantly reduced latency times in the passive avoidance test (Fig. 1a). NXP032-treated aged mice exhibited a significant increase in spontaneous alternation percentage compared to vehicle-treated aged mice (*p* < 0.05). In the Y-maze test, aged mice showed a decrease in short-term working memory compared to young mice (Fig. 1b). NXP032-treated mice showed a significant increase in latency times compared to vehicle-treated mice (*p* < 0.001). NXP032 can effectively prevent memory impairment in aged mice.

### NXP032 decreases aging-induced microvascular damage in the cortex

**Figure 2:**
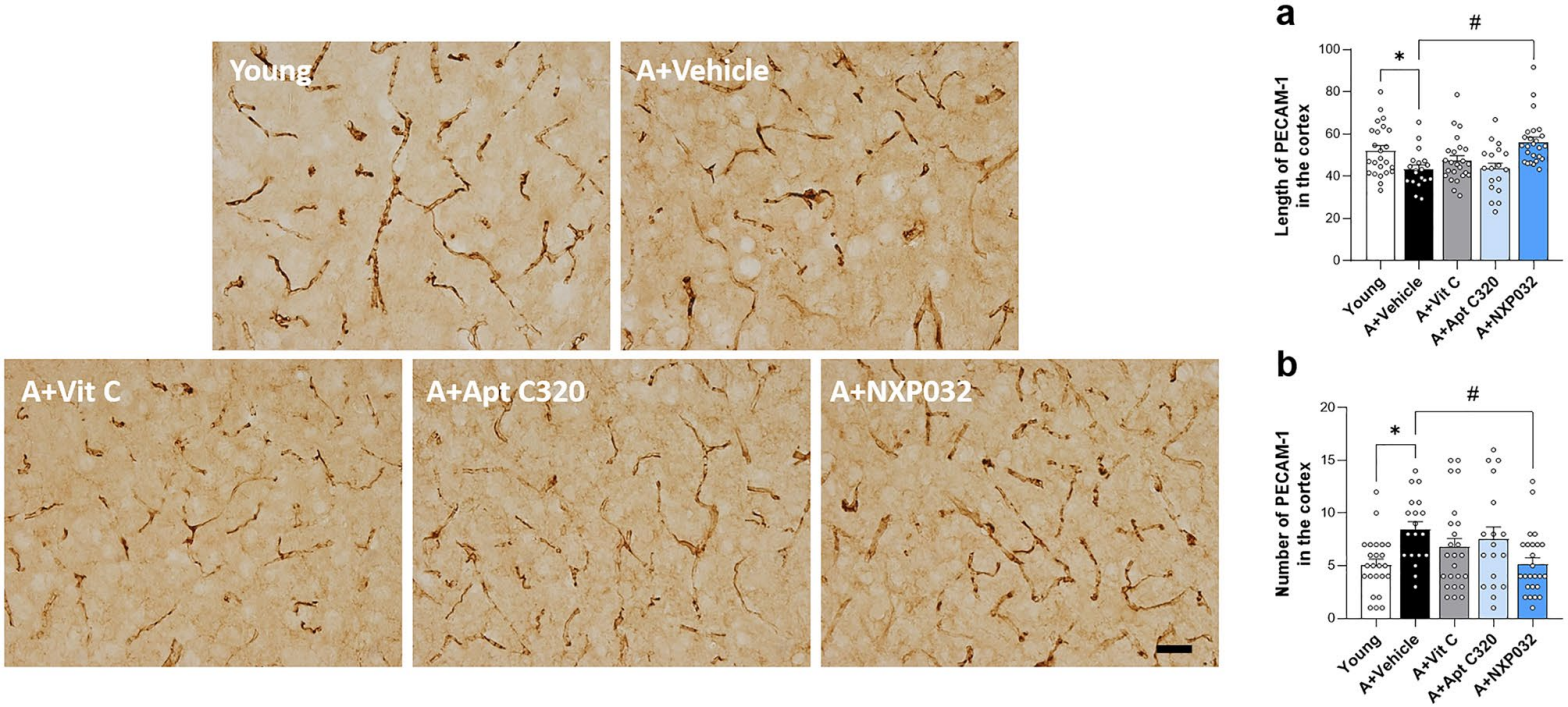
NXP032 treatment improved microvessel damage in aged mice, as evidenced by representative photographs of PECAM-1 immunostaining in the cortex region. (**a**) Aged mice exhibited a decrease in the length of microvessels, but treatment with NXP032 increased the length of microvessels. (**b**) Aged mice showed an increase in the number of microvessels less than 10 μm compared to young mice, but treatment with NXP032 reversed this effect. Data represent the mean ± standard error of the mean. *p < 0.05, compared to the young group. ^#^*p* < 0.05, compared to the A + Vehicle group. Scale bar: 50 μm.

Platelet endothelial cell adhesion molecule-1 (PECAM-1) is expressed by all endothelial cells, including those in the microvascular compartment of the brain. PECAM-1 was used as an endothelial cell marker to measure the length of microvessels and fragments^16^. PECAM-1 immunostaining is shown in Fig. 2. We observed significant differences among the groups in the microvessels fragmentation (Fragmentation, F = 5.650, *p* < 0.001; Length, F = 3.613, *p* < 0.01), fragmented microvessels were measured less than 10 μm. The microvessels of aged mice showed narrower, shorter, and more irregular patterns in the cortex than young mice (*p* < 0.01). NXP032-treated mice showed a reduction in the fragmentation of microvessels (*p* < 0.01) and an increase in their length (*p* < 0.05) compared to aged mice. These results indicate that NXP032 treatment alleviated the damage to microvessels induced by aging.

### NXP032 reduces the BBB damage in the cortex

**Figure 3:**
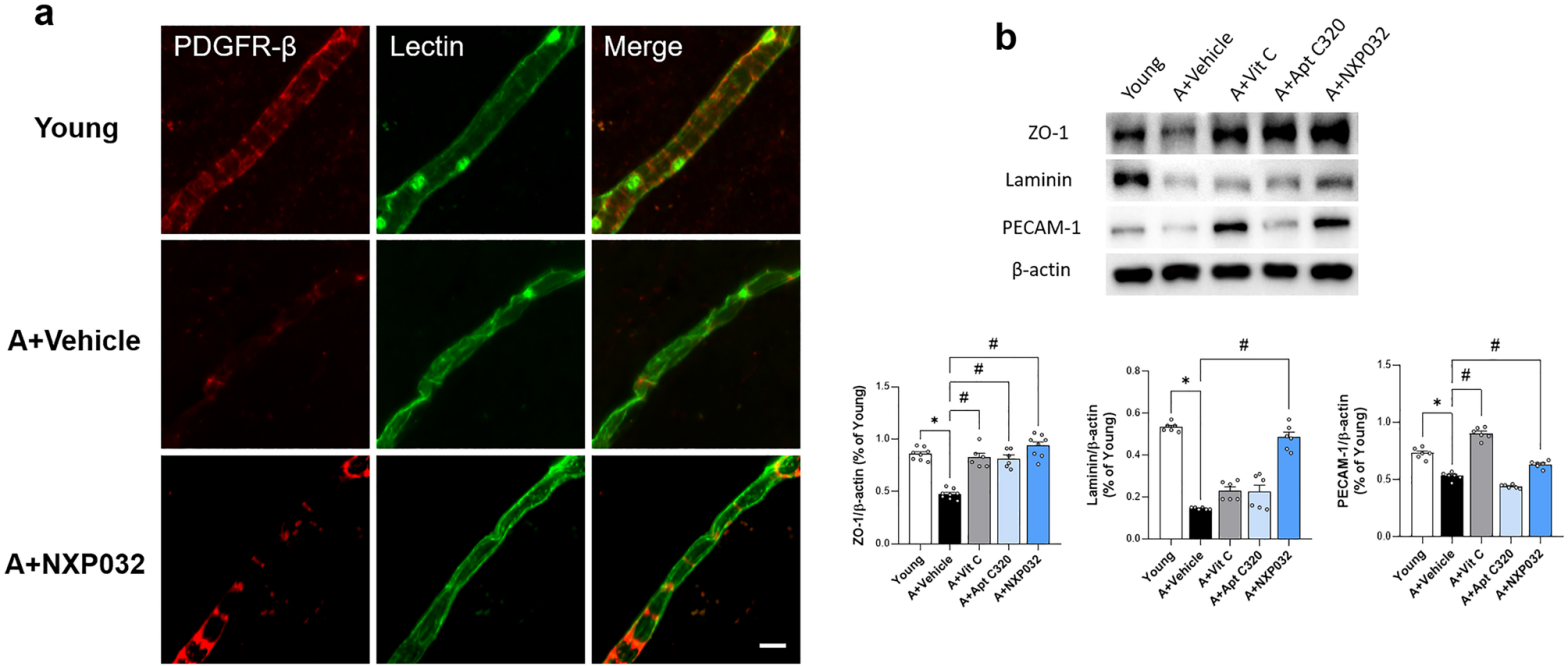
NXP032 treatment ameliorates BBB disruption in aged mice. Representative photographs of immunostaining of PDGFR-β and lectin expression in the cortex region. (**a**) Aged mice exhibited decreased PDGFR-β expression in the cortex compared to young mice, but NXP032 treatment increased PDGFR-β expression. (**b**) Representative photomicrographs and quantitative analysis of western blot analysis of ZO-1, laminin, and PECAM-1 proteins expression in the cortex. Aged mice had decreased key components of neurovascular units in the cortex, but NXP032 treatment restored these components. Data represent the mean ± standard error of the mean. *p < 0.05, compared to the young group. ^#^*p* < 0.05, compared to the A + Vehicle group. Scale bar: 10 μm.

BBB damage occurs in the aging brain^17^. To confirm whether NXP032 reduced BBB damage, we identified the expression of platelet-derived growth factor receptor-β (PDGFR-β), zonula occludens-1 (ZO-1), and laminin as a critical component of the basement membrane. PDGFR-β is a receptor expressed by pericytes, which are crucial for maintaining BBB integrity and brain homeostasis^18^. Decreased PDGFR-β expression has been linked to various neurodegenerative diseases and is associated with BBB damage^19^. As shown in Fig. 3a, the expression of PDGFR-β was reduced in the brains of aged mice, and treatment with NXP032 increased PDGFR-β expression. ZO-1 is one of the major tight junction proteins that form the BBB, and its decreased expression due to aging increases BBB permeability^20^. Laminin is also a component of the basal membrane that makes up the BBB, and its decreased expression during aging is linked to increased BBB permeability^21^. We confirmed the levels of ZO-1, laminin, and PECAM-1 expression using a western blot, and there were statistically significant differences among the groups in Fig. 3b (ZO-1, F = 38.256, *p* < 0.001; Laminin, F = 71.685, *p* < 0.001; PECAM-1, F = 133.746, *p* < 0.001). The expression levels of ZO-1, laminin, and PECAM-1 were significantly lower in aged mice than in young mice (ZO-1, *p* < 0.001; laminin, *p* < 0.001; PECAM-1, *p* < 0.001). But, NXP032-treated mice significantly increased the levels of the ZO-1, laminin, and PECAM-1 proteins compared to aged mice (ZO-1, *p* < 0.001; laminin, *p* < 0.001; PECAM-1, *p* < 0.001). The increase in PDGFR-β, ZO-1, and laminin expression in NXP032-treated mice may suggest a potential therapeutic benefit for the normal aging process associated with BBB damage.

### NXP032 ameliorates the activation of glial cells and string vessels in the hippocampus

**Figure 4:**
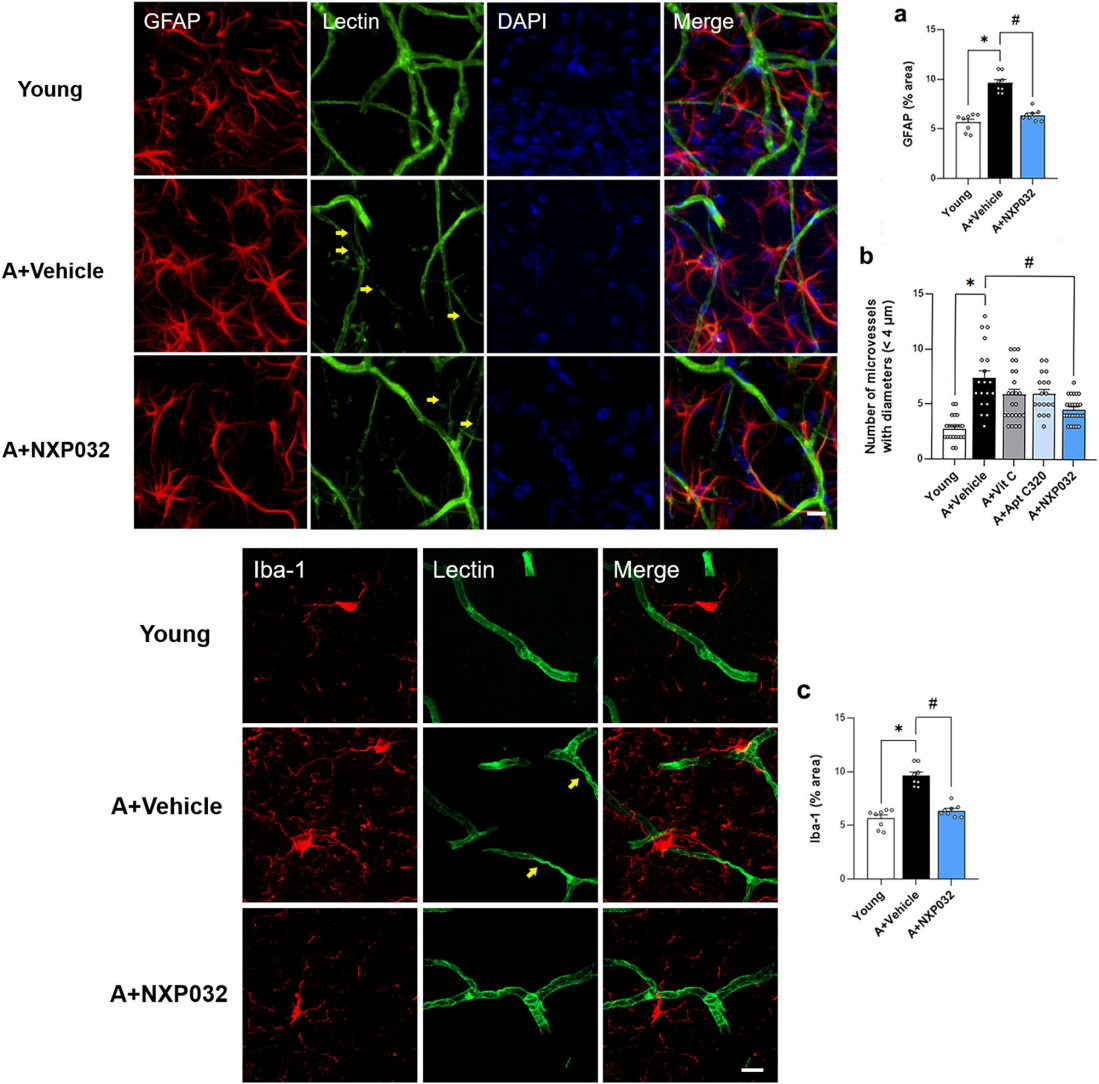
NXP032 treatment ameliorates activation of glial cells in the hippocampal CA1 region in aged mice. Representative photographs of GFAP and Iba-1 immunostaining and lectin expression in the hippocampal CA1 region. (**a**) Aged mice exhibited increased GFAP expression compared to young mice, but NXP032 treatment decreased GFAP expression. (**b**) Yellow arrows indicate string vessels. (**c**) Aged mice increased activated Iba-1 expression compared to young mice, but NXP032 treatment decreased Iba-1 expression. Data represent the mean ± standard error of the mean. *p < 0.05, compared to the young group. ^#^*p* < 0.05, compared to the A + Vehicle group. Scale bar: 10 μm.

To investigate whether NXP032 ameliorates neuroinflammation in the brain, we checked astrocytes and microglia expression using immunofluorescence (Fig. 4). Aged mice exhibited a significant increase in glial fibrillary acidic protein (GFAP) and ionized calcium-binding adaptor molecule 1 (Iba-1) expression in the hippocampal CA1 region compared to young mice (F = 51.951, *p* < 0.001; F = 54.537, *p* < 0.001) (Fig. 4a,c). Also, microglial and astrocytic activation is accompanied by distinct morphological alterations, including thickened processes, increased branching, and cell body enlargement. NXP032 treatment reduced the activation of glial cells, showing a pattern similar to that of young mice (GFAP, *p* < 0.001; Iba1, *p* < 0.001). These results indicate that NXP032 reduces neuroinflammatory reactions in the hippocampal CA1 of aged mice.

With aging, decreased blood flow and occlusion contribute to endothelial cell death, capillary collapse, and the formation of string vessels^22^. String vessels are a term used to describe a type of blood vessel that appears as a thin and filamentous structure. These vessels are characterized by their lack of endothelial cells and narrow diameter (typically less than 4 μm)^23^. To analyze the string vessels in the brain, microvessels with a diameter less than 4 μm were measured (Fig. 4b). There was a significant difference in the number of microvessels in the hippocampal CA1 among the five groups. The number of microvessels with diameters less than 4 μm was found to be higher in aged mice than in young mice (F = 15.939, *p* < 0.001). NXP032-treated mice showed a decreased number of microvessels with a diameter of less than 4 μm compared to aged mice (*p* < 0.001), which suggests NXP032 can alleviate microvessel damage.

## Discussion

Even in elderly individuals without dementia, cognitive decline and impairment are noticeable with age^24^. The functional capacity of the brain gradually decreases during aging, resulting in decrements in learning and memory, attention, decision-making speed, sensory perception, and motor coordination^25,26^. These aspects of cognitive decline are among the biggest health threats in old age and are manifested by a loss of brain function^27^. Many animal experiments have reported that aged rodents have decreased learning and memory ability in the Morris water maze and elevated plus maze tests compared to young rodents^28^. Consistent with previous studies, our study found that 20-month-old mice had impairments in spatial learning and memory, but the NXP032 group showed an increased spontaneous rate in the Y-maze test and a significant increase in the passive avoidance test (Fig. 1). Based on these results, we confirmed that NXP032 can improve cognitive impairment due to aging.

Although age-related cognitive impairment is caused by many factors, we focused on damage to the neurovascular unit in this study^29^. Aging-induced increases in oxidative stress have profound effects on various components of the NVU^30^. Specifically, damage of NVU is resulting in decreased microcirculatory blood flow in the brain. In our study, aged mice exhibited narrower, shorter, and more irregular microvessel construction in the cortex (Fig. 2). Furthermore, we found that the expression of the pericyte marker, PDGFR-β, the tight junction protein, ZO-1, and the basal lamina protein, laminin, were significantly reduced in aged mice. However, NXP032 treatment reduced aging-induced BBB disruption and disarranged microvessels in the aged brain (Fig. 3). Another study reported an age-related decrease in the expression of ZO-1 in brain capillaries of Pdgfrβ+/− mice compared to controls, with a reduction of 40–50% and 80% in 6–8- and 14–16-month-old mice, respectively. Furthermore, the study compared the expression of ZO-1 in Pdgfrβ+/− mice and F7 mutants with that of controls and found significant reductions of 48% and 37%, respectively, in 6- to 8-month-old mice^31^. Therefore, it appears that damage to ZO-1 is closely associated with Pdgfrβ. Based on our results, we suggest that treatment with NXP032 can improve the age-related reduction in ZO-1 and PDGFR-β, thereby alleviating damage to the BBB.

Pericytes are specialized cells closely associated with the endothelial cells that make up the blood vessels in the brain. They play a critical role in maintaining the structural integrity of the blood vessels and the proper functioning of the BBB^32^. Adequate pericyte coverage is thought to be critical for maintaining the structural integrity of the blood vessels and the proper functioning of the BBB^33^. Loss of pericytes or decreased pericyte coverage has been associated with BBB dysfunction and increased permeability^34^. PDGFR-β is a receptor protein that is expressed on the surface of pericytes, and it is used as a marker for identifying and quantifying pericytes in the brain. Detection of PDGFR-β immuno-reactivity is a commonly used method for evaluating pericyte coverage in the brain^32^. We showed the results of PDGFR-β immunostaining in Fig. 3a, and NXP032 treatment alleviated the decrease in PDGFR-β expression due to aging. The disruption of the BBB and alterations in the expression of tight junction proteins are associated with oxidative stress in the aged brain^35^. These results indicate that NXP032 may alleviate microvascular damage and BBB destruction in the brain.

Morphological changes in the neurovascular unit are prominent in aging mouse models, showing reduced microvessel density and altered shape of small blood vessels^8^. These changes increased small vessel torsion and string vessels in various brain regions^36^. There have been reports of increased string vessels in Alzheimer’s disease compared to age-matched controls associated with vascular damage due to aging^37^. In this study, we found that tortuosity and string of small vessels were detected in the brains of aged mice, but these abnormal vessels were significantly more frequent in the aged group than in the NXP032-treated group (Fig. 4).

Microglia and astrocytes interact with pericytes in the central nervous system (CNS) and play essential roles in the immune system, homeostasis, angiogenesis, BBB maintenance, and synaptic support^38,39^. Normal microglia and astrocytes are responsible for directing the formation, maturation, and clearance of synapses as well as ion homeostasis^40^. Aging increases the secretion of cytokines through the activation of microglia, which, in turn, promotes the activity of astrocytes^41^. This process is widely recognized as a hallmark of neuroinflammation, leading to proinflammatory cytokine release, neurotoxicity, neuronal dysfunction, and BBB destruction^42^. We confirmed that the expression of GFAP and Iba-1 in the cortex and hippocampal CA1 regions was activated in aged mice compared to young mice. Interestingly, NXP032-treated mice showed reduced expression of activated GFAP and Iba-1, showing a pattern similar to that of young mice (Fig. 4).

Aptamers are short, single-stranded RNA/DNA molecules capable of binding to specific target molecules with high affinity and specificity^43,44^. Aptamers have a wide range of potential applications in human health, including diagnostics, therapeutics, and research tools. In terms of translation to human use, aptamers have already been developed for various diagnostic and therapeutic applications, including cancer, anticoagulation therapy, and virus detection. Some aptamer-based medicines have even received regulatory approval for use in humans. One example is pegaptanib, an aptamer that targets vascular endothelial growth factor and is used to treat macular degeneration^45^. NXP032 has been deemed safe, as evidenced by its recent GRAS (Generally Recognized as Safe) approval by the FDA. For therapeutic development, Nexmos is currently conducting a separate study focusing on the pharmacokinetics (PK), pharmacodynamics (PD), and safety of NXP032.

Our study has several limitations. First, our aged mice model only included female mice. Males and females typically have different lifespans and frequently differ in their responses to neuroprotective mechanisms, immunity, and drug metabolism^46,47^. Therefore, it is necessary to investigate the effects of aging and NXP032 on male mice. Second, pharmacokinetic and toxicity evaluations for NXP032 were not included. Toxicity evaluation may indicate the safety of NXP032. Third, direct evaluation of vascular damage was not presented in this study. Leakage of BBB induced by damage to NUV can be identified using dextran or Evans blue, which are direct methods to check the integrity of the BBB. Unfortunately, these tests were not conducted in this study, so we could not obtain the clear results. This is one of the limitations of this study. Therefore, it is necessary to apply an evaluation method that can directly confirm BBB damage in future experiments. If these limitations are supplemented, the mechanism of action of NXP032 on neurovascular aging is expected to be more complete.

NXP032 is a potent antioxidant that maximizes the function of ascorbic acid and seems to maintain cognitive function by ameliorating BBB disruption and glial activation in aged mice. Considering that there are no effective drugs for age-related cognitive impairment, we suggest that NXP032 has the potential as a new therapeutic in improving age-related vascular damage.

## Methods

### Animals and drug treatments

Female C57BL/6 6-week-old (19 ± 3 g, Orient Bio Inc., Seongnam, Gyeonggi-do, Republic of Korea) and female C57B216J 17-month-old (40 ± 10 g, Korea Basic Science Institute, Buk-gu, Gwangju, Republic of Korea) mice were used for the present study. Animals were housed where water and food were freely consumed, and the environment was regulated (12 h light/dark cycle; 23 ± 2 °C; humidity of 50 ± 10%). This study was approved by the Animal Care Committee of Kyung Hee University (KHSASP-20-113), and the authors complied with the ARRIVE (Animal Research: Reporting of In Vivo Experiments) guidelines and the Korean Academy of Medicine Animal Care Guidelines. Aptamers are short strands of oligonucleotides or peptide molecules that can bind to specific target molecules and modulate their activity^43,44^. Using Nexmos Co., Ltd.’s self-developed SELEX (Systematic Evolution of Ligands by Exponential enrichment) technology, it specifically binds to vitamin C (ascorbic acid) as a single-stranded DNA aptamer, allowing its influx into the brain and prolonging the antioxidant effect in the brain. The purified DNA aptamer NXP032 (ascorbic acid/DNA Aptamin C320 complex) was dissolved in distilled water at 95 °C for 5 min and then cooled slowly at room temperature to form a tertiary structure. Aptamin C320 was mixed with ascorbic acid (Sigma‒Aldrich, St. Louis, MO, USA) at a ratio of 1:50 (*w/w*) to produce final NXP032 (200 mg/kg ascorbic acid and 4 mg/kg Aptamin C320).

In the experimental design, 6-week-old female mice and 17-month-old female mice were randomly divided into 5 groups (n = 40): The young group (6-week-old mice), the A + Vehicle group (17-month-old mice + vehicle treatment), the A + Vit C group (17-month-old mice + vitamin C treatment), the A + Apt C320 group (17-month-old mice + Aptamin C320 treatment), and the A + NXP032 group (17-month-old mice + NXP032 treatment). Mice were treated with vehicle, 200 mg/kg vitamin C, 4 mg/kg Aptamin C320, and NXP032 by oral gavage for 8 weeks simultaneously every day. After 8 weeks of treatment, cognitive behavioral experiments were conducted for 2 weeks. All mice were sacrificed the next day after the cognitive behavioral tests were finished.

### Passive avoidance test

The passive avoidance test is a fear-aggravated test used to assess learning and memory in rodent models of CNS disorders^48^. The manual evacuation system consists of two chambers separated by a wall with a guillotine door. During the training phase (1 day), mice acclimatized to the environment in the dark chamber for 60 s, and then when the light was turned on, they naturally moved to the dark chamber according to their instincts. When the mouse moves, the guillotine closes and the mouse receives an electric shock (0.5 mA for 2 s) through the stainless grid floor. The same procedure was performed in the retention test (2 days), and the waiting time for the mice to enter the dark room was measured for 300 s.

### Y-maze test

The Y-maze test is a test to evaluate short-term memory and spatial perception^15^. In the Y-maze test apparatus, each arm made of wood is divided into three branches, and each branch is 25 cm long, 14 cm high, and 5 cm wide at the same angle (120° apart). After designating each arm as A, B, and C, the mice were allowed to move freely for 6 min starting from the center, and then the movements of the mice were recorded. Mice were considered to have entered only when their hind legs had passed more than half of the maze when entering each arm. Changed behavior was assessed by recording the order in which each arm was entered. Only one point was recognized when entering each arm consecutively (alternation, ABC, ACB, BAC, BCA, CAB, or CBA). However, no points were awarded if each arm did not enter consecutively (for example, ABA, ACA, BAB, BCB, CAC, or CBC). The spontaneous alternation (%) was calculated by the following formula: Spontaneous alteration (%) = [(total of number of alternations)/(total arm entries − 2)] × 100.

### Cortex and hippocampus tissues preparation and immunohistochemistry

After the experiment was complete, we ensured that the animal was deeply anesthetized by a trained researcher following approved protocols and guidelines. Once the animal was under deep anesthesia, it was euthanized by exsanguination, which involved draining all of its blood. Mice were sacrificed under deep anesthesia, perfused with 50 mM phosphate buffered saline (PBS), fixed with 4% paraformaldehyde (PFA), and brains were removed. Brain tissues were extracted and fixed in 4% PFA for 1 day and then fixed in 30% sucrose solution in PBS for 4 days. Using a freezing microtome (CM3050S, Leica Biosystems GmbH, Nussloch, Germany), the brain was frozen sectioned to a thickness of 30 μm in the coronal direction. After blocking endogenous peroxidase with 3% H_2_O_2_ in PBS for 20 min at room temperature, brain tissues were then processed using a Mouse on Mouse detection kit (M.O.M. kit®, Vector Laboratories, Burlingame, CA, USA) to remove nonspecific labels using mouse monoclonal antibodies on mouse brain tissue. Brain tissues were blocked with 1% bovine serum albumin (BSA) and 10% normal goat serum in PBS for 2 h. Brain tissues were then incubated overnight with PECAM-1 (1:1000, LSBio, LifeSpan BioSciences, Seattle, WA, USA) at 4 °C. Brain tissues were incubated with anti-mouse secondary antibody (1:200, Vector Laboratories, Burlingame, CA, USA) for 2 h at room temperature. The bound secondary antibody was amplified using the antibody-biotin-avidin-peroxidase complex solution (Vector Elite ABC kit®; Vector Laboratories, Burlingame, CA, USA) for 1 h at room temperature. Brain tissues were visualized using 3.3′-diaminobenzidine tetrahydrochloride (DAB kit; Vector Laboratories, Burlingame, CA, USA) for 5 min. Brain tissues were mounted onto gelatin-coated slides and air-dried overnight at room temperature. Coverslips were mounted using Permount® (Vector Laboratories, Burlingame, CA, USA). In the cortical region, the number of microvessels and fragments less than 10 μm in length were counted in several places using Image-Pro® Plus software (Media Cybernetics, Inc., Rockville, MD, USA) for quantitative analysis.

### Immunofluorescence

Lycopersicon esculentum (Tomato) lectin, DyLight 488 (Thermo Fisher Scientific, Waltham, MA, USA), was slowly injected with intracardiac perfusion (100 µg/mL) for 20 s. Brain tissues were washed and blocked with 3% BSA for 2 h at room temperature. They were then incubated overnight at 4 °C with goat antibody to GFAP (1:500; Santa Cruz Biotechnology, CA, USA), rabbit antibody to PDGFR-β (1:500, Abcam, Cambridge, UK), and rabbit antibody to Iba-1 (1:500, Abcam, Cambridge, UK). Brain tissues were washed and incubated with Alexa Fluor 594-conjugated donkey anti-rabbit IgG and Alexa Fluor 594-conjugated goat anti-mouse IgG (1:1000; Molecular Probes, Eugene, OR) for 90 min at room temperature. Brain tissues were then washed and mounted onto gelatin-coated slides. The coverslips were mounted using 4′,6-diamidino-2-phenylindole. Fluorescence staining was observed using confocal microscopy (LSM 700, Zeiss, Oberkochen, Germany).

### Western blotting

Proteins were extracted from mouse brain tissue using radioimmunoprecipitation assay (RIPA) buffer (Thermo Fisher Scientific, Waltham, MA, USA). Proteins were separated on 8–10% SDS gels and transferred to polyvinylidene difluoride (PVDF) membranes. Western blot images were generated by cutting out individual portions of the whole membrane and incubating them with the respective antibodies after excision. The membrane was blocked with 5% skim milk at room temperature for 1 h, then anti-ZO-1 (1:200, Lifespan Biosciences, WA, USA), anti-laminin (1:1000, Abcam, Cambridge, UK), PECAM-1 (1:1000, LSBio, LifeSpan BioSciences, Seattle, WA, USA) and β-actin (1:10,000, Santa Cruz, CA, USA) were incubated overnight at 4 °C. The membrane was washed with PBST, and membranes were incubated with a 1:2000 dilution of horseradish peroxidase-conjugated anti-rabbit and mouse secondary antibodies for 2 h. Band detection was performed using chemiluminescence (Clarity™ Western ECL Substrate, Bio-Rad, Hercules, CA, USA) on the membrane. Analysis was performed using ImageJ to compare relative protein expression.

### Statistical analysis

All data are expressed as the mean ± standard error of the mean (S.E.M). Statistical analysis was performed using SPSS software version 26.0 (IBM corp., Chicago, IL, USA). Differences among groups were analyzed by one-way ANOVA, followed by Tukey's test for multiple comparisons. *P* values of < 0.05 were considered statistically significant.

### Institutional review board

The study was conducted according to The Kyung Hee University Guidelines for Institutional Animal Care and Use Committee (protocol code KHSASP-20-113).

## Supplementary Information

Supplementary Information 1.

Supplementary Information 2.

Supplementary Information 3.

Supplementary Information 4.

Supplementary Information 5.

Supplementary Information 6.

## Data Availability

All data generated or analysed during this study are included in this published article and its Supplementary Information files.
