# Supplementary figures and images for: NXP032 ameliorates cognitive impairment by alleviating the neurovascular aging process in aged mouse brain
Source: Sci Rep. 2023 May 26;13:8594. doi: 10.1038/s41598-023-35833-x (PMC10219997; doi:10.1038/s41598-023-35833-x)

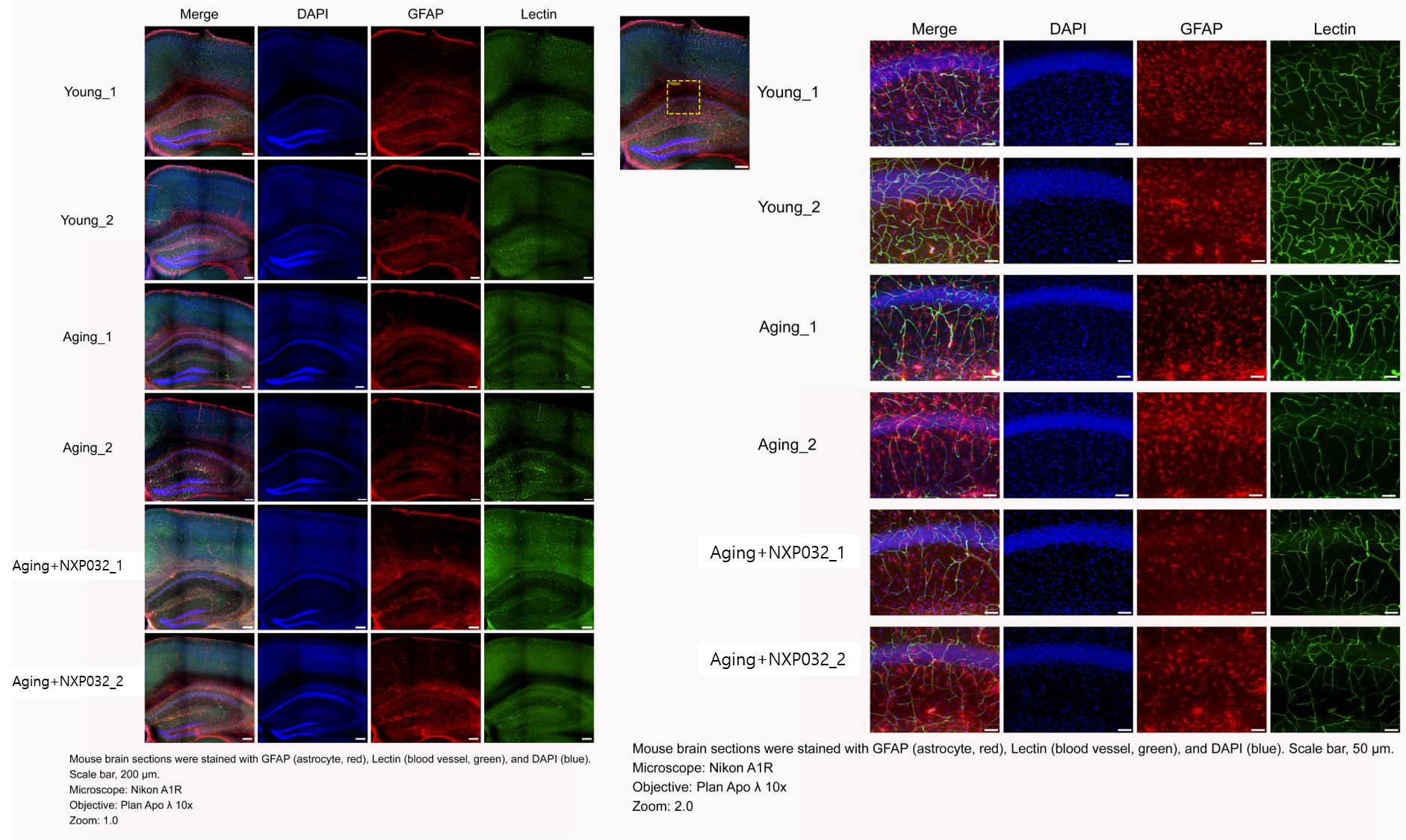

Supplement: Supplementary file 1 — Supplementary Information 1. [file 41598_2023_35833_MOESM1_ESM.tif]
