# Supplementary material for: NXP032 ameliorates cognitive impairment by alleviating the neurovascular aging process in aged mouse brain
Source: Sci Rep. 2023 May 26;13:8594. doi: 10.1038/s41598-023-35833-x (PMC10219997; doi:10.1038/s41598-023-35833-x)
Supplement: Supplementary file 6 — Supplementary Information 6. [file 41598_2023_35833_MOESM6_ESM.docx]

List of Supplementary information.

Supplementary Figure 1. Full gel pictures

Supplementary Table 1. List of primary antibodies

Supplementary Figure 1. Full gel pictures


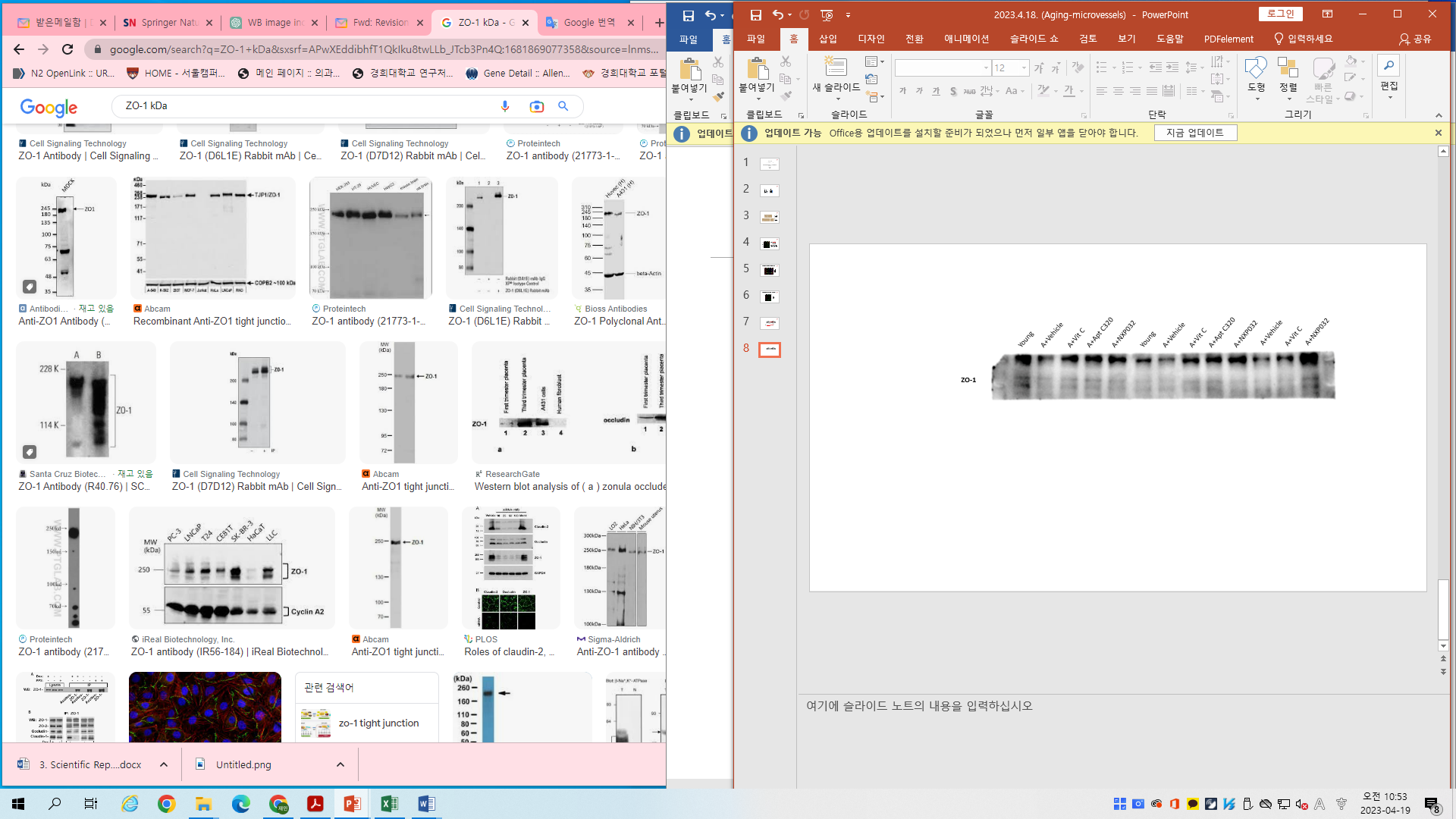


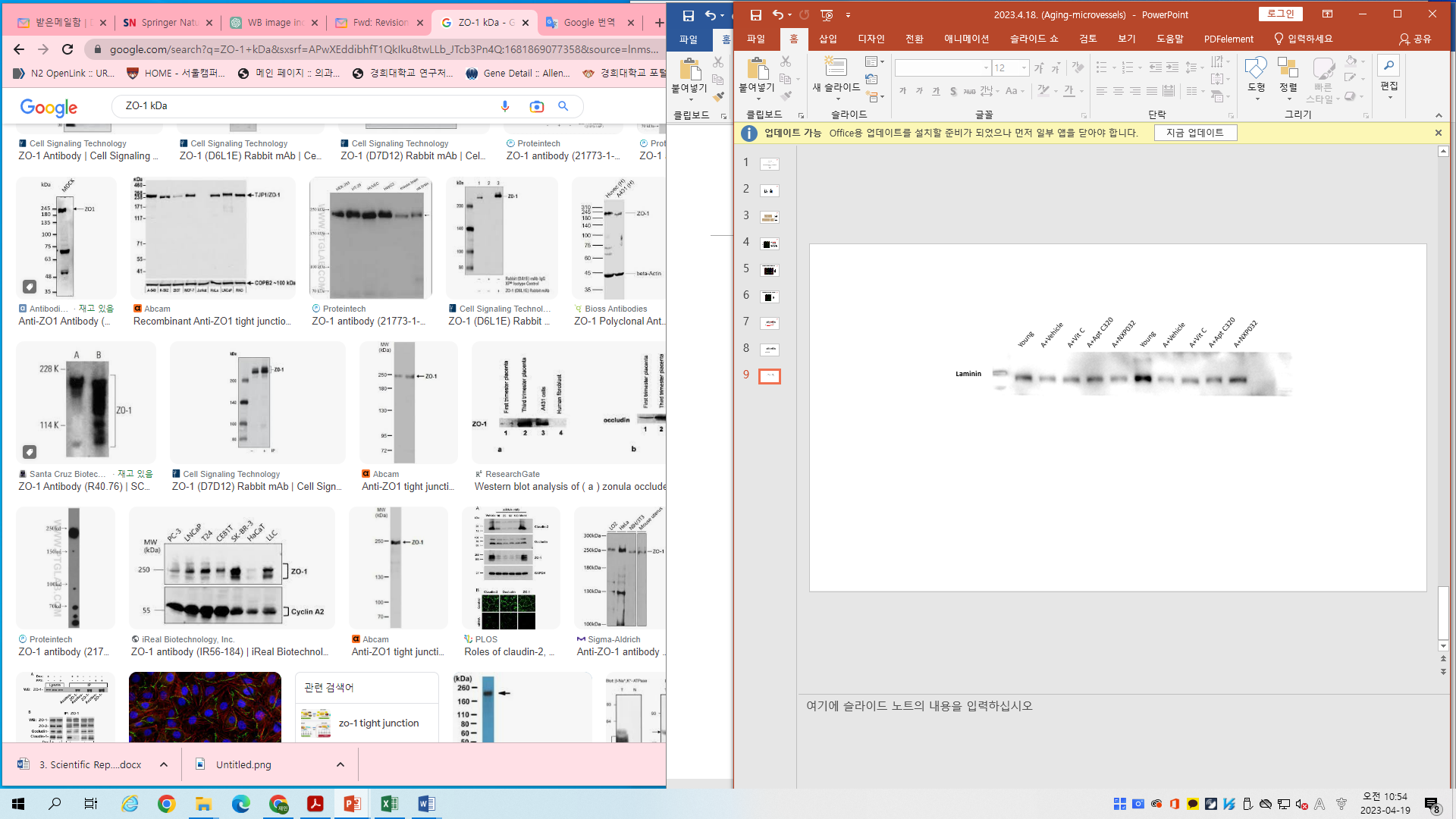


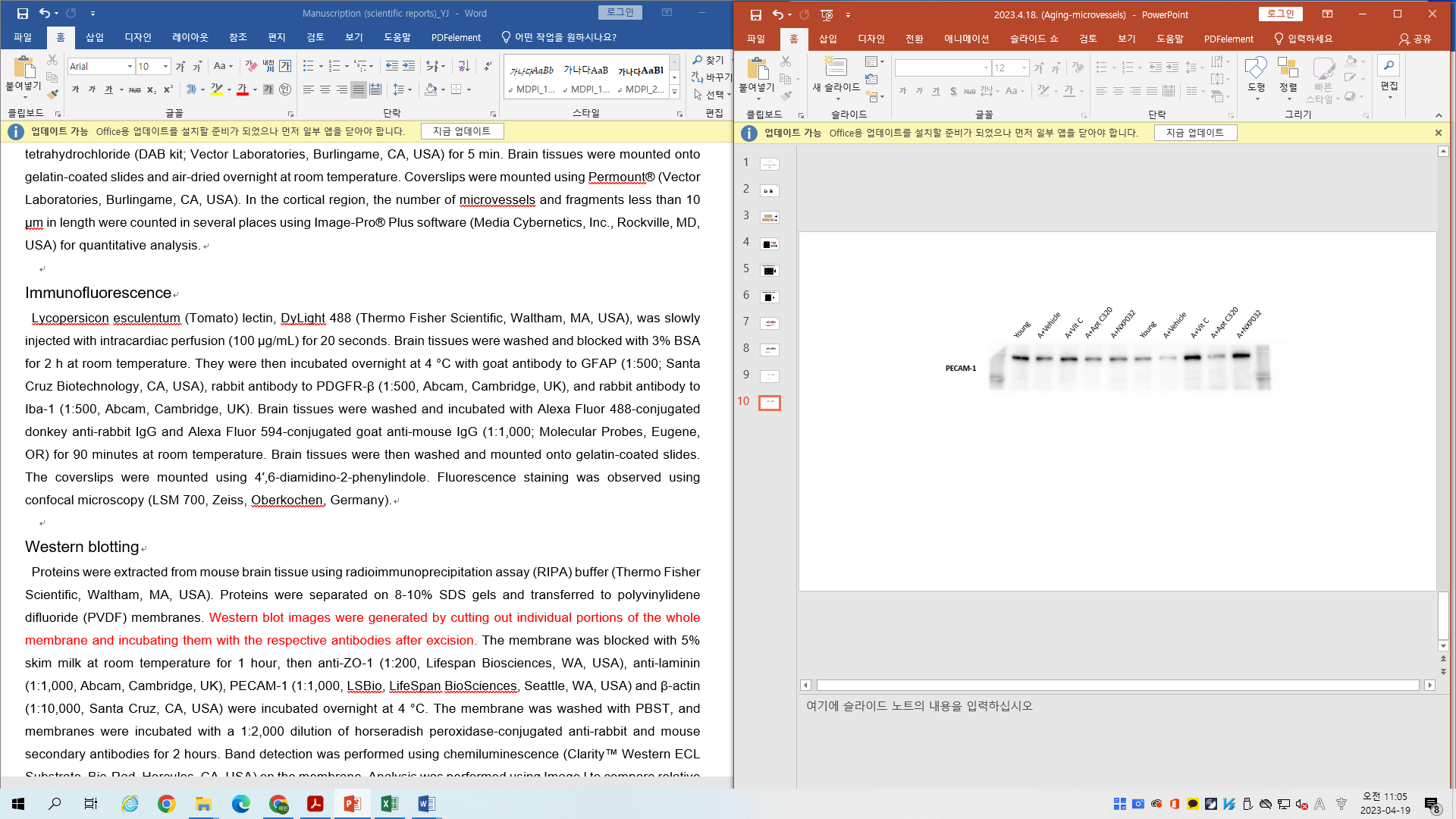


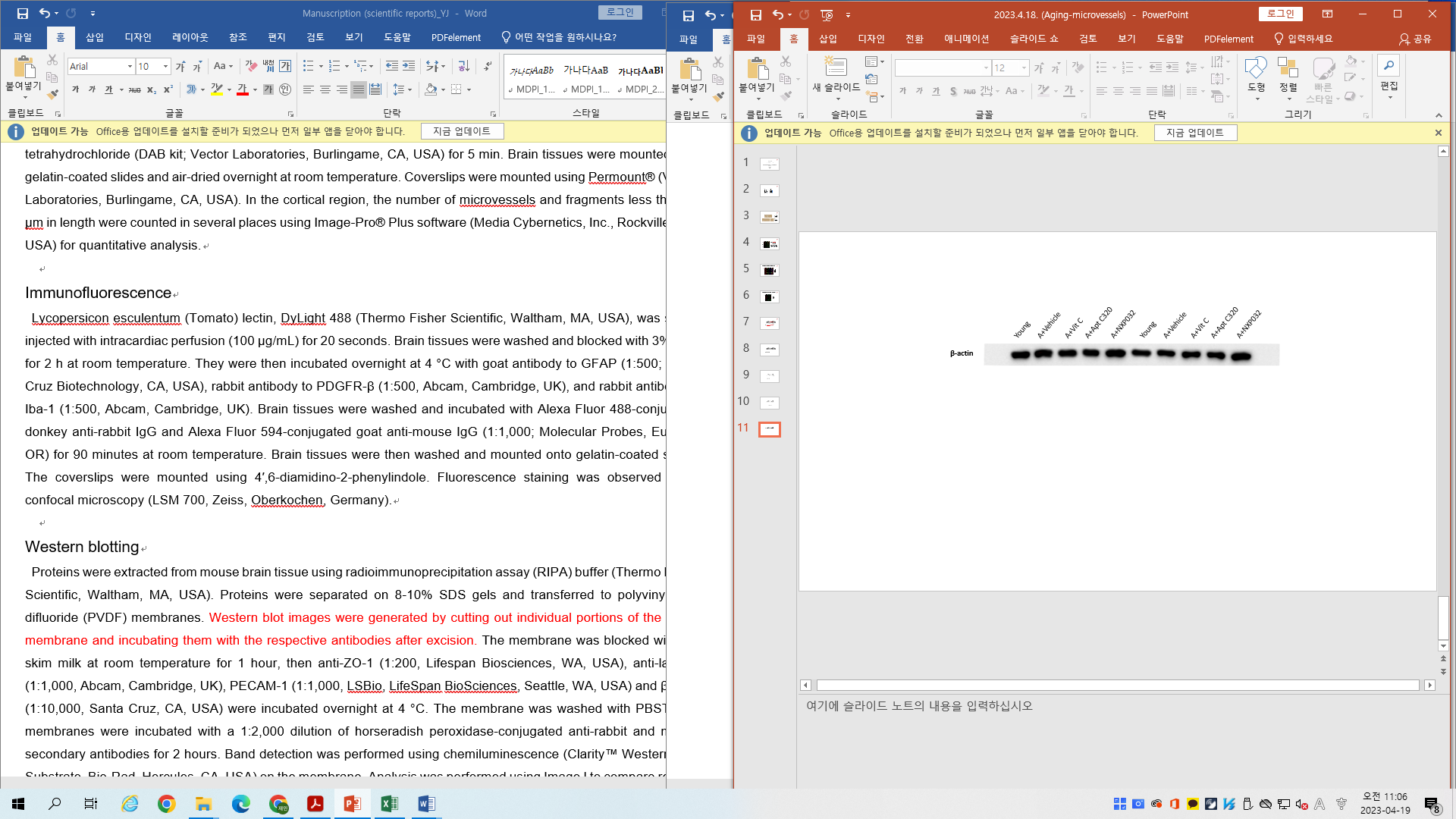


Supplementary Table 1. List of primary antibodies

| Antigen | Source | Catalog# | Dilution |
| --- | --- | --- | --- |
| ZO-1 | LSBio | LS-B2129 | 1:200 |
| PECAM-1 | LSBio | LS-C34736 | 1:1,000 |
| laminin | Abcam | Ab-11575 | 1:1,000 |
| β-actin | Santa Cruz | Sc-47778 | 1:10,000 |
| GFAP | Santa Cruz | Sc-1670 | 1:500 |
| PDGFR-β | Abcam | Ab32570 | 1:500 |
| Iba-1 | Abcam | Ab178846 | 1:500 |
